# Supplementary figures and images for: SARS-CoV-2 spike E156G/Δ157-158 mutations contribute to increased infectivity and immune escape
Source: Life Sci Alliance. 2022 Mar 16;5(7):e202201415. doi: 10.26508/lsa.202201415 (PMC8927725; doi:10.26508/lsa.202201415)

Cell lysate

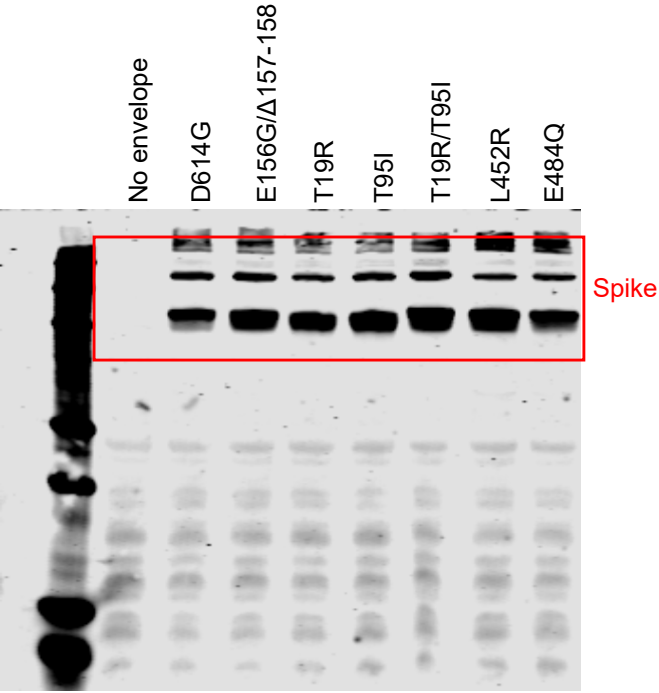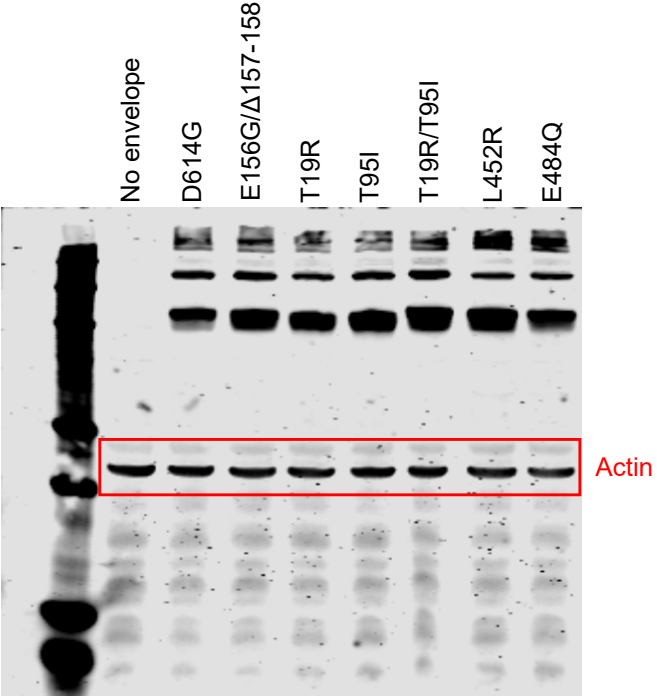

Viral lysate

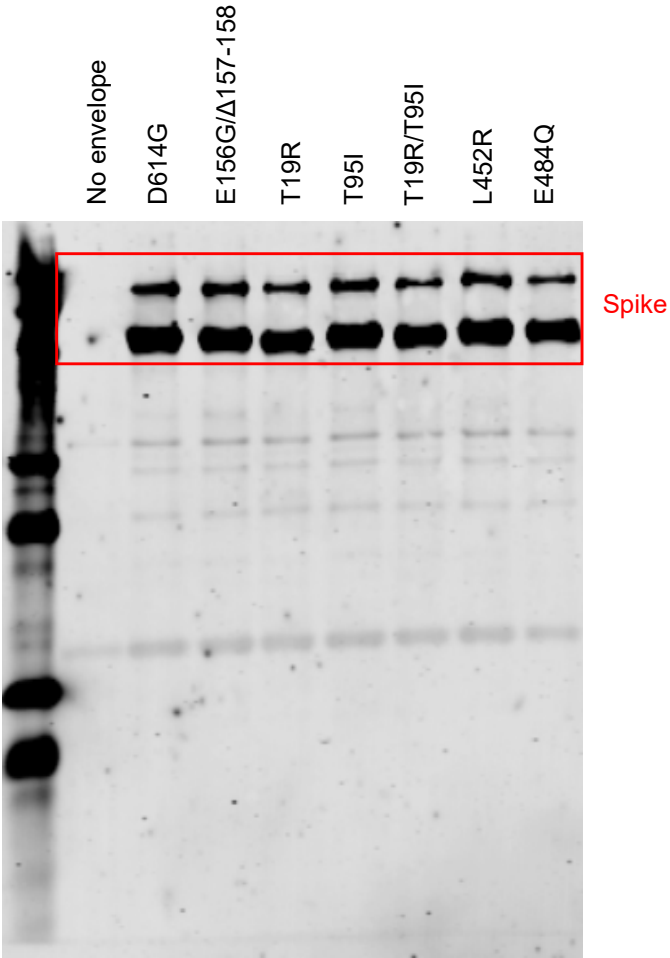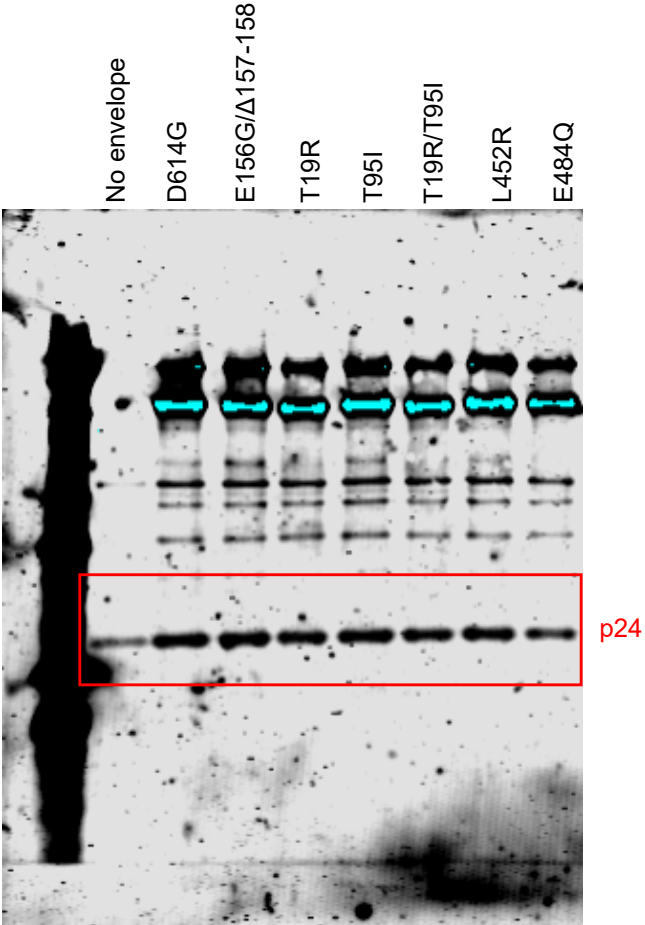

Supplement: Supplementary file 1 [file LSA-2022-01415_SdataF2.pdf]

Cell lysate

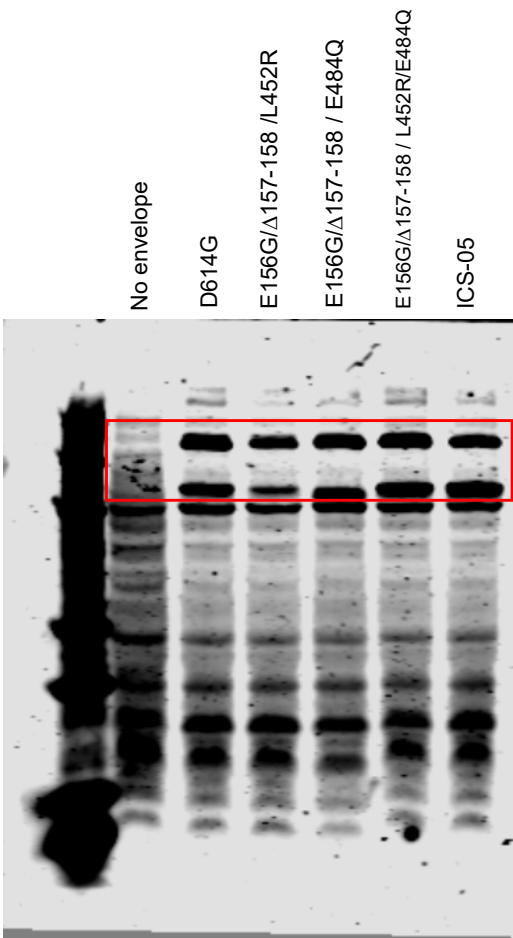

Spike

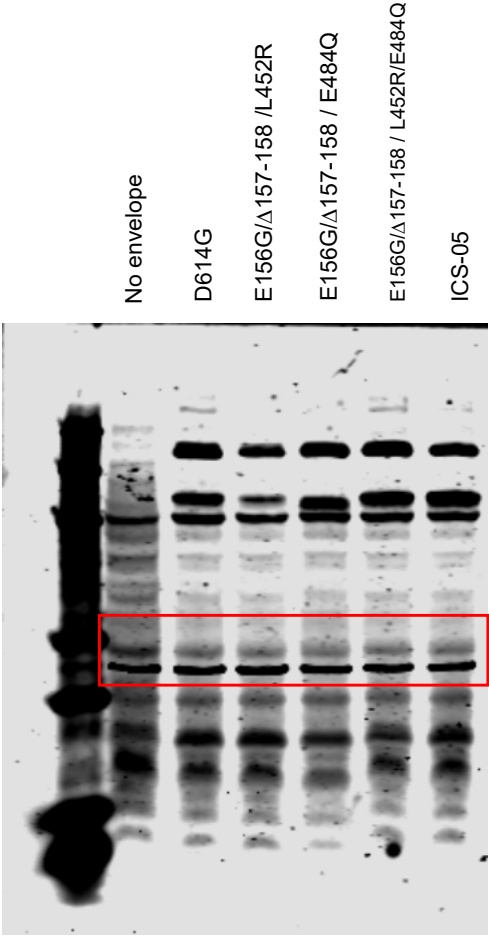

Actin

Viral lysate

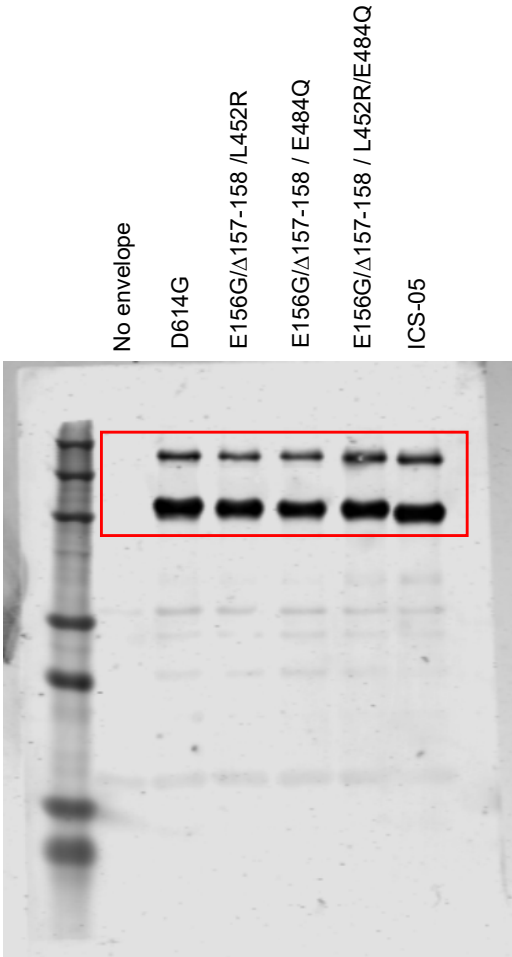

Spike

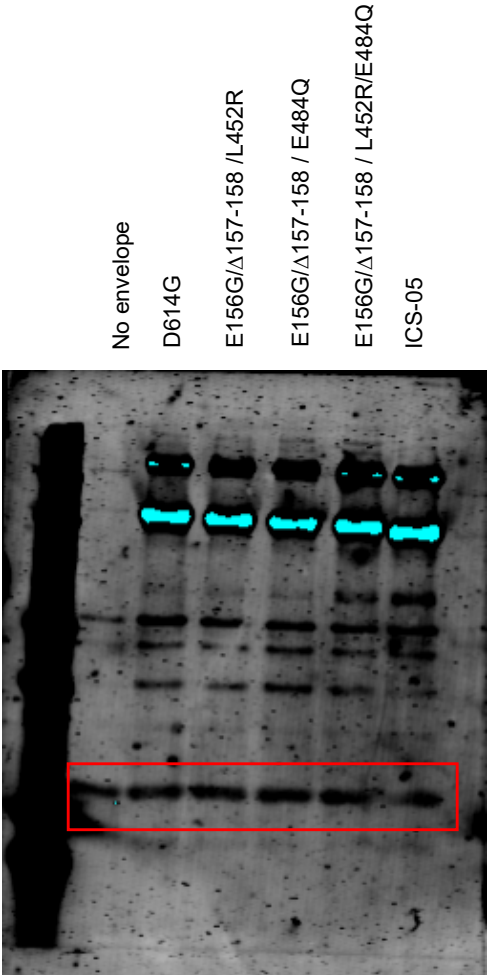

p24

Supplement: Supplementary file 2 [file LSA-2022-01415_SdataF3.pdf]
