# Supplementary material for: SARS-CoV-2 spike E156G/Δ157-158 mutations contribute to increased infectivity and immune escape
Source: Life Sci Alliance. 2022 Mar 16;5(7):e202201415. doi: 10.26508/lsa.202201415 (PMC8927725; doi:10.26508/lsa.202201415)
Supplement: Supplementary file 4 [file LSA-2022-01415_TableS1.pdf]

Table S1: List of plasmids used in this study

S. No.	Plasmid Name	Purpose	Notes	Source/Described in
1.	pScalps Luciferase Zsgreen	Expression of firefly luciferase and Zoanthus green fluorescent protein	Lentiviral vector with luciferase gene under SFFV promoter and Zsgreen under Cyclophilin promoter	Mishra et. al. 2021
2.	Spike del19 codon-optimized in pcDNA3.1(-)	Expression of mammalian codon optimized SARS CoV-2 spike glycoprotein	A nineteen amino acid deletion at the C terminal	Addgene (#155297), from Raffaele De Francesco's lab
3.	psPAX2	Lentiviral packaging plasmid	Packaging vector	Addgene (#12260) from Didier Trono's lab
4.	pMD2.G	Expression of VSV-G glycoprotein	Envelope plasmid	Addgene (#12259) from Didier Trono's lab
5.	pScalps Hygro	Lentiviral vector backbone for selection of transduced cells by hygromycin	Hygromycin resistance marker and an MCS for expression under SFFV promoter	This study
6.	pcDNA3.1BS(-)	Control for transfection and vector backbone for cloning	Mammalian expression vector	Mishra et al 2021
7.	ACE2 in pScalps Hygro	Establishment of ACE2+ cell line	ACE2 expression construct	Mishra et. al. 2021
8.	N protein in pcDNA3.1BS(-)	Expression of SARS-CoV-2's nucleocapsid protein	2X Strep Tag	Mishra et. al. 2021

9.	pcDNA3.1BS(-) Spike D614G	Spike mutant expression	Spike D614G mutant expressing plasmid	Mishra et. al. 2021
10.	pcDNA3.1BS(-) Spike T19R/D614G	Spike mutant expression	Spike T19R/D614G mutant expressing plasmid	This study
11.	pcDNA3.1BS(-) Spike T95I/D614G	Spike mutant expression	Spike T95I/D614G mutant expressing plasmid	This study
12.	pcDNA3.1BS(-) Spike T19R/T95I/D614G	Spike mutant expression	Spike T19I/T95I/D614G mutant expressing plasmid	This study
13.	pcDNA3.1BS(-) Spike E156G/Δ157-158/D614G	Spike mutant expression	Spike E156G/Δ157-158/D614G mutant expressing plasmid	This study
14.	pcDNA3.1BS(-) Spike L452R/D614G	Spike mutant expression	Spike L452R/D614G mutant expressing plasmid	This study
15.	pcDNA3.1BS(-) Spike E484Q/D614G	Spike mutant expression	Spike E484Q/D614G mutant expressing plasmid	This study
16.	pcDNA3.1BS(-) Spike E156G/Δ157-158/L452R/D614G	Spike mutant expression	Spike E156G/Δ157-158/L452R/D614G mutant expressing plasmid	This study
17.	pcDNA3.1BS(-) Spike E156G/Δ157-158/E484Q/D614G	Spike mutant expression	Spike E156G/Δ157-158/E484Q/D614G mutant expressing plasmid	This study
18.	pcDNA3.1BS(-) Spike E156G/Δ157-158/L452R/E484Q/D614G	Spike mutant expression	Spike E156G/Δ157-158/L452R/E484Q/D614G mutant expressing plasmid	This study

19.	pcDNA3.1BS(-) Spike ICS-05	Spike mutant expression	Spike ICS-05 expressing T19R/T95I/ E156G/Δ157-158/L452R/E484Q/D614G mutant expressing plasmid	This study
