# Supplementary material for: SARS-CoV-2 spike E156G/Δ157-158 mutations contribute to increased infectivity and immune escape
Source: Life Sci Alliance. 2022 Mar 16;5(7):e202201415. doi: 10.26508/lsa.202201415 (PMC8927725; doi:10.26508/lsa.202201415)
Supplement: Supplementary file 5 [file LSA-2022-01415_TableS2.pdf]

Table S2: List of Reagents used in this study

**Reagent**	**Company**	**Catalog No.**
Dulbecco’s Modified Eagle Medium (DMEM)	Biowest, USA	L0102
Fetal Bovine Serum (FBS), Certified, Performance tested. Origin: United States	Gibco, USA	10082-147
L-Glutamine	Gibco, USA	25030-081
Pen-Strep (Penicillin Streptomycin)	Gibco, USA	15140-122
Hoechst 33342	Sigma Aldrich	14530
PBS	HyClone, USA	SH30256.02
Poly-L-Lysine	Sigma Aldrich	P4832- 50ML
EMPARTA ACS Sucrose	Merck	1.94921.1021
Tris(2-carboxyethyl) phosphine hydrochloride (TCEP)	Sigma Aldrich	75259
2xcOmplete^{TM}, EDTA-free Protease inhibitor cocktail	Sigma Aldrich	11873580001
PVDF membrane	Immobilon-FL, Merck-Millipore	IPFL00010
Blocking Buffer	Sigma Aldrich	WBAVDFL01
Beta-Actin Rabbit Monoclonal Antibody	LI-COR Biosciences	926-42210
IRDye 680RD Goat anti-Mouse IgG antibody	LI-COR Biosciences	925-68070
IRDye 800CW Goat anti-Rabbit IgG antibody	LI-COR Biosciences	925-32211
Anti-HIV-1 P24 antibody	NIH-ARP 366	N/A
